# Physicochemical Characterization and Oxidative Potential of Iron-Containing Particles Emitted from Xuanwei Coal Combustion

**DOI:** 10.3390/toxics11110921

**Published:** 2023-11-11

**Authors:** Senlin Lu, Jin Liu, Guoqing Hou, Jiumei Zhao, Xinchun Liu, Tingting Xie, Kai Xiao, Shinichi Yonemochi, Enyoh Christian Ebere, Weiqian Wang, Qingyue Wang

**Affiliations:** 1 School of Environmental and Chemical Engineering, Shanghai University, Shanghai 200444, China; 18406405830@shu.edu.cn (J.L.); hou_guoqing@shu.edu.cn (G.H.); jmzhao1999@163.com (J.Z.); xietingting3683@163.com (T.X.); 2 Institute of Desert Meteorology, China Meteorological Administration, Urumqi 830002, China; 3 College of Urban and Environmental Sciences, Beijing University, Beijing 100871, China; 2206391517@pku.edu.cn; 4 Centers for Environmental Science in Saitama, Saitama 374-0115, Japan; yonemochi.shinichi@pref.saitama.lg.jp; 5 School of Science and Engineering, Saitama University, Saitama 338-8570, Japan; enyoh.c.e.527@ms.saitama-u.ac.jp (E.C.E.); weiqian@mail.saitama-u.ac.jp (W.W.); seiyo@mail.saitama-u.ac.jp (Q.W.)

**Keywords:** iron-containing minerals, size-resolved particles, hydroxyl free radical, oxidation potential

## Abstract

Respiratory diseases have been proven to be directly related to air pollutants. Xuanwei, located in South China, has been known to have the highest mortality rate for lung cancer in China because of the air pollutants emitted through local coal combustion. However, the mechanism of lung cancer induced by air pollutants is not clear. Based on the fact that a large number of iron-bearing mineral particles was found in Xuanwei coal combustion particles, the iron-containing particles were hypothesized to play important roles in the pathogenesis of the high incidence rate of lung cancer in this area. In this study, raw coal samples were collected from a coal mine in the Xuanwei area. Size-resolved particles emitted from the raw coal samples were collected using an Anderson high-volume sampler. Mineralogical characterization and an assessment of the oxidative potential of the iron-containing particles were conducted using cutting-edge technologies, and the biological activity of the particles were evaluated via DTT assay. Our data showed that the iron-containing minerals accounted for more than 10% of the measured particles emitted from Xuanwei coal combustion samples. The content analysis of ·OH generated from Xuanwei coal combustion particles showed that ·OH content was dependent on the size of particles in the surrogated lung fluid. The concentration of ·OH increased as the particle size decreased. The DTT assay data further demonstrated that when the mass concentration of dissolved irons increased, the oxidation potential of the particles increased. The highest proportion of divalent iron in the total dissolved iron was found in the submicron particles in low pH solution(pH = 1), which indicated that the oxidative potential induced by submicron particles was stronger than that induced by coarse particles and fine particles. Armed with the above data, the toxicological mechanism of the iron-containing mineral particles can be investigated further.

## 1. Introduction

Air pollutants can increase the risk of lung cancer. Xuanwei, located in the southeast of China, has the highest incidence and mortality rate of lung cancer in the country [1]. Women who live in this area have the highest incidence of lung cancer among non-smokers in China. High lung cancer rates have been reported in Xuanwei since the 1980s [2]. The prevalence of lung cancer was believed to be associated with local bituminous coal combustion emission pollutants [3]. However, the etiology of lung cancer in the region remains unclear. Several risk factors, such as toxic metals [4,5], polycyclic aromatic hydrocarbons (PAHs) [6], and nano crystal SiO_2_ [3,4] have been reported to have relationship with the development of lung cancer in Xuanwei residents. More recently, researchers believed that the high content of nano mineral particles emitted during coal combustion might contribute to the high incidence rate of lung cancer [7], and nanosized particles were also found in the pulmonary and normal tissues of lung cancer patients using TEM-EDX [8]. Importantly, ambient particles (mainly from local coal combustion) in the Xuanwei atmosphere have the ability to produce free radicals, whose toxicity poses a potential risk to human health [4]. The limited data on this topic have demonstrated that mineral particles in the Xuanwei atmosphere might play a role in the lung cancer. Although the mineral particles are mainly distributed among coarse particles (PM_10–2.5_), a large number of nano-sized mineral particles can still be found in fine (PM_2.5_) and submicron particle fractions [7]. As one of the dominant transition metals, iron has been reported in ambient particles regarding its solubility, speciation, and mineralogy. Importantly, these iron-containing particles could remain suspended in the atmosphere for hours or even up to two weeks and pose risks to human health and the ecological system [9,10].

Normally, iron exists in crystalline oxides in the form of Fe^2+^ or Fe^3+^. As the iron-containing particles are inhaled into human lungs, the valence between Fe^2+^ and Fe^3+^ allows them to transform into each other through Fenton reactions, and simultaneously, free radicals can be released. In addition, reactive oxygen species (ROS) formed by iron-containing particles, could actually promote the uptake of more particles by cells, causing continuous and increasingly serious damage to the human lungs.

However, few papers have focused on the oxidative potential caused by iron-containing particles emitted from Xuanwei coal combustion; therefore, we collected raw coal samples from a coal mine, stimulated the burning process, and sampled the particles using a high-volume sampler. Then, physicochemical characterization and an assessment of the oxidative ability of the iron-containing particles were conducted. We tried to provide fundamental data for explaining the high incidence of lung cancer in Xuanwei residents.

## 2. Materials and Methods

### 2.1. Coal Burning Experiment and Coal Combustion Particle Sampling

The raw coal samples were collected from Guangming Coal Mine (latitude, 26°19′46.551″; longitude 104°09′36.435″) in Laibin Town, Xuanwei. The raw coal belonged to bituminous coal, which lies in the uppermost portion of the Xuanwei Formation (Upper Permian) [11]. The coal combustion system has been described previously by Lu et al. (2016) [7]. Briefly, 500 g of raw coal was ignited and then moved to a stove, which was placed in a closed flared fume hood. During the coal combustion, clean air was introduced through the blower (50 m^3^/min) and mixed with the flue gas. The flue gas temperature in the chamber was around 40 °C. To ensure the reliability of the experimental data, the particulate matter in the air was filtered with a filter cloth at the inlet of the blower, which can minimize background pollution during sampling.

Size-resolved particles from the coal combustion were sampled using a high-volume air sampler (Shibata Science Co., Ltd., Saitama, Japan) at a flow rate of 566 L/min. The particles with aerodynamic diameters were <1.1 μm (regarded as submicron particles), 1.1–2.0 μm (fine particles), 2.0–3.3 μm, 3.3–7.0 μm (coarse particles), and >7.0 μm could be collected. For individual particle analysis, the ambient particles were sampled onto lacey carbon films supported by Cu grids using a multi-nozzle cascade impact (MCI) sampler at a flow rate of 20 L/min.

### 2.2. Chemical Element Analysis of the Coal Combustion Particles

An energy-dispersive X-ray fluorescence spectrometer Epsilon 4 (ED-XRF, Malvern Panalytical, B.V. Almelo, The Netherlands) was employed for the chemical element analysis of the coal combustion samples. The method has been previously described in detail by Shaltout et al. (2020) [12]. A blank quartz filter was used as a control. The concentration of each chemical element measured in the blank filter was used as the base value for determining the concentration of each element in the combustion particle samples.

### 2.3. Detection of Environmentally Persistent Free Radicals (EPFRs) and OH Radicals in the Coal Combustion Particles

To identify EPFRs, quartz fiber filters were cut into strips, moved into a quartz EPR tube, and measured via electron spin resonance spectroscopy. The relevant parameters were as follows: sweep time, 120 s; center field, 324.74 mT; sweep width, 25 mT; modulation frequency, 100 kHz; modulation width, 0.05 mT; microwave frequency, 9105.26 MHz; and microwave power, 0.998 mW. More information can be found in our previous work [5]. For ·OH radical detection, 10.0 mmol/L sodium benzoate (NaBA) was employed as a prober to measure ·OH radicals. Hydroxybenzoic acid (ρ-HBA) was formed after the NaBA reacted in the solution with radicals extracted from coal combustion particles, and the ρ-HBA could be measured via HPLC. After 0.5 mL particle solution was filtered through 0.22 μm filter, the fraction was transferred to a HPLC injection bottle. Then, 100.0 μmol/L deferoxamine mesylate (DSF) and 50.0 μmol/L HSO^3−^ were added to end the reaction, and the solution was kept for 10 min under dark. Then, 5.0 μL H_2_SO_4_ (1.0 mol/L) was added to allow for OH detection via HPLC. The mass concentration of ·OH was calculated as follows:

(1) $$\left[\cdot OH]=[\rho \mathrm{-HBA}]/(Y_{\rho \mathrm{-HBA}}\times \xi_{BA}\right)$$

where [ρ-HBA] is the yield of ρ-HBA from the standard curve. The value of the $Y_{\rho \mathrm{-HBA}}$ in the SLF (surrogated lung fluid) is the yield ratio of BA, as described previously [13,14], and *ξ*_BA_ is the fraction of the ascorbic acid that reacts with the BA (0.9972) [15].

### 2.4. Identification of Iron-Containing Particles

The elemental composition of the individual particles was determined semi-quantitatively using a JEM-2010 TEM (JEOL, Japan). The TEM was coupled with an energy-dispersive X-ray spectrometer (EDX) that could detect elements heavier than carbon. Carbon and copper were not considered in the chemical element analysis because of the effects of the carbon-coated film on the TEM grid [16]. EDX spectra were collected for only 30 s to minimize the radiation exposure and potential beam damage.

### 2.5. Iron Dissolution from the Coal Combustion Particles

The iron dissolution measure protocol was described in our previous work [17,18]. Briefly, the acid solution (0.1 mol/L HCl, HNO_3_, 0.05 mol/L H_2_SO_4_, pH = 1) was purged with N_2_ for 30 min before adding the samples. All reactions were performed at 25 °C without oxygen and under dark conditions. The reactors were placed in a water bath to maintain a constant temperature. Then, an appropriate volume of the suspension was obtained from the flask, filtered through a 0.22 μm PTFE filter, and immediately acidified to a final concentration of 0.2 mol/L of HCl. Dissolved Fe (II) was measured using the 1,10-phenanthroline chromogenic method after acidification (Stucki, 1981). To analyze the concentration of Fe (II), 50 μL of 0.43 mol/L NH_4_F, 500 μL of 5 mmol/L 1,10-phenanthroline, and 1 mL of ammonium acetate buffer were added to the acidified solution and mixed thoroughly. For FeT, 500 μL 1.5 mol/LNH_2_OH·HCl, 500 μL 5 mmol/L 1,10-phenanthroline, and 1 mL ammonium acetate buffer were added to the sample. The absorbance values of the sample solution were measure at 510 nm using a T6 UV–Vis spectrophotometer and used to calculate the concentration of Fe. The FeT included both dissolved Fe (II) and dissolved Fe (III), and the solubility of the Fe(II) and Fe(III) were calculated using the following formulae:

Fe(II) solubility = Dissolved Fe(II)/Total Fe × 100% (2)

Fe(III) solubility = Dissolved Fe(III)/Total Fe × 100% (3)

### 2.6. Oxidative Potential of Coal Combustion Particles

A measure of 1.75 ml of diluted dissolved solution was fully mixed with 0.5 mL of 0.5 mol/L chelex treated potassiumphosphate buffer solution (pH = 7.4), and 0.25 mL of 1.5 mmol/L dithiothreitol (DTT) was added at 37 °C [19]. After an interval, 100 μL of the reaction solution was collected and blended with 0.5 mL 1% *w*/*v* trichloroacetic acid to terminate the reaction between the DTT and dissolved solution. This was then mixed with 0.25 mL of 0.3 mmol/L dithio dinitrobenzoic acid (DTNB) and 1 mL of 0.12 mol/L Tris-HCl buffer solution (pH = 8.9), containing 6 mmol/L of edetic acid (EDTA). After homogenous mixing, a 200 μL solution was collected to measure the absorbance at 405 nm using a microplate reader (Bio-Rad, iMark™, Hercules, CA, USA). DTT consumption was calculated as follows:

(4) $$\mathrm{DTT}\;\mathrm{consumption}\;{\mathrm{rate}}_{\mathrm{blank}\;\mathrm{or}\;\mathrm{sample}}\;({\mathrm{nmolmin}}^{-1})=-\;\sigma \mathrm{Abs}\cdot \frac{N_{0}}{\mathrm{Ab}s_{0}};$$

(5) $$\mathrm{DTT}\;\mathrm{activity}\;(\mathrm{nmolmi}n^{-1}mg^{-1})=\frac{\mathrm{DTT}\;\mathrm{consumption}\;{\mathrm{rate}}_{\mathrm{sample}}-\mathrm{DTT}\;\mathrm{consumption}\;{\mathrm{rate}}_{\mathrm{blank}}}{m_{particles}}$$

where −σAbs denotes the slope of absorbance as a function of time; Abs0 represents the absorbance calculated from the intercept of the linear regression; N0 (nmol) denotes the initial moles of DTT added into the reaction vial; and m_particle_ (mg) is the mass of particles used in the reaction vial.

### 2.7. Statistical Analysis

Statistical analysis was conducted using SPSS software version 11.5 (SPSS, Inc., Chicago, IL, USA). The data were obtained from three independent experiments and presented as mean ± standard error of the mean (SEM).

## 3. Results

### 3.1. Percentage of Iron-Containing Particles among the Measured Particles

We once reported that the main minerals from Xuanwei coal combustion were quartz (SiO_2_), hematite (Fe_2_O_3_), sulfates (CaSO_4_), and aluminates [7]. The EDX spectrum of showed that the Fe element could be identified, along with other chemical elements such as Al, Ca, S, etc. Based on the quantitative results of the EDX analyses and a previous report [20], the weight ratio of iron-containing particles was found to be higher than 65%.

P(Fe) = Fe/(Na + Mg + Al + Si + S + Cl + K + Ca + Ti + Fe).(6)

**Figure 1:**
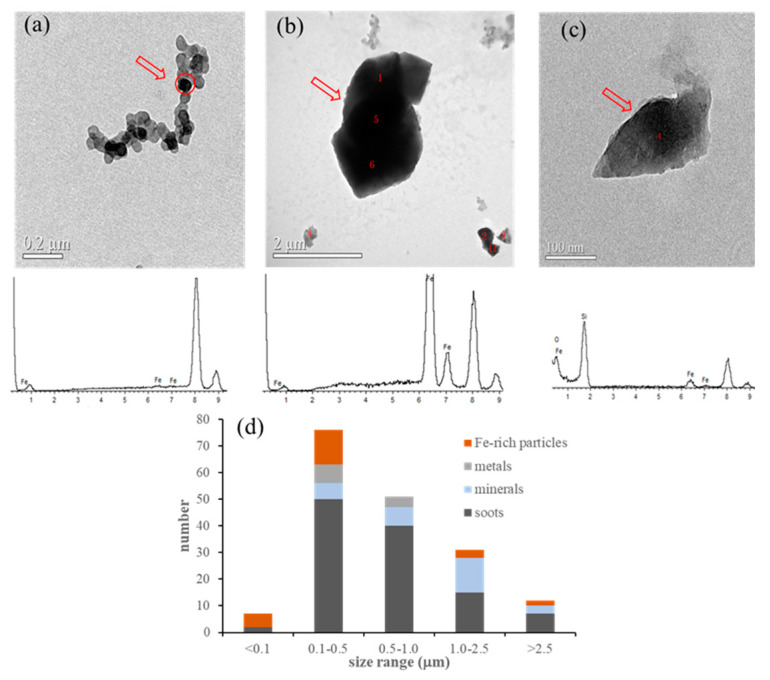
Microscopic characterization and size distribution of Fe–containing particles from Xuanwei coal combustion. (**a**) Fe–containing particles with soots; (**b**,**c**) Fe–rich minerals; (**d**) size distribution of Xuanwei coal combustion particles.

Among the 576 particles found, 51 were identified as iron-containing particles. The size of the Fe-containing particles ranged from 0.1 to 0.5 μm (Figure 1d).

### 3.2. Intensity of Free Radicals Generated by Xuanwei Coal Combustion Particles

**Figure 2:**
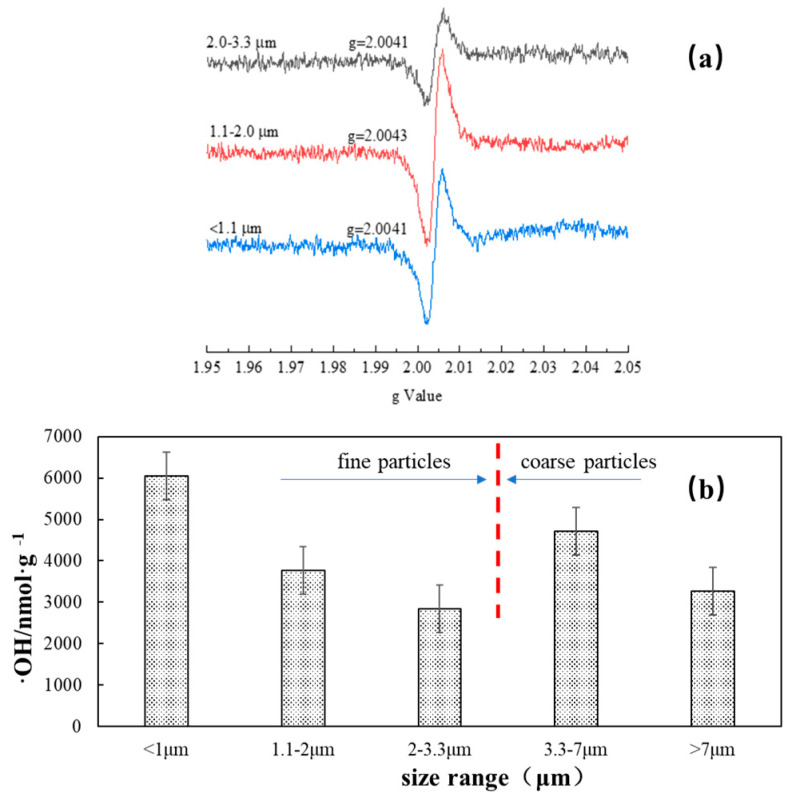
Free radicals generated by the size-resolved particles from Xuanwei coal combustion. (**a**) Free radicals measured by ESR; (**b**) mass levels of the hydroxyl radicals from the size-resolved particle solution measured via HPLC.

We once reported that the Xuanwei atmosphere contained the following ambient particles, in order of their free radical ability: fine particles > coarse particles > submicron particles [4]. In this study, we found that the free radical intensity of the 1.1–2.0 um particles (grade 3) (g = 2.0043) was stronger compared with that of coarse particles and submicron particles (g = 2.0041) (Figure 2a). Previous studies have suggested that g factors >2.0040 are typical for oxygen-centered radicals [21].

The content analysis of ·OH generated from Xuanwei coal combustion particles showed that the ·OH content was dependent on particle size in the SLF (surrogated lung fluid) solution after 24 h (Figure 2b). The highest mass level of the hydroxyl radicals was found in the solution of submicron particles, and the concentration of ·OH increased in both fine particles (<1 μm, 1.1–2 μm, 2–3.3 μm) and coarse particles (3.3–7 μm, >7 μm) as the particle size decreased.

### 3.3. Chemical Elements in the Size-Resolved Particles from Xuanwei Coal Combustion

The presence of chemical elements in the coal combustion particles was measured via XRF (Table 1). Na, Mg, Al, K, Ca, V, Mn, Fe, Co, Cu, and Zn were the main metallic elements. Iron was mainly distributed in the coarse particles (size > 7.0 μm) with the highest mass level of 2976.69 μg/g, followed by 2045.68 μg/g (3.3–7.0 μm), 1399.80 μg/g (2.0–3.3 μm), 1187.81 μg/g (1.2–2 μm), and 263.17 μg/g (<1.0 μm). However, the individual data showed that the highest number of Fe-containing particles was found in the size range 0.1–0.5 μm. The XRF and TEM data clearly demonstrated that Fe-containing particles from Xuanwei coal combustion could be found across the different size ranges. It was noted that submicron particles could be enriched in some of the elements, such as S, Cl, Br, and Pb.

### 3.4. Dissolution Ratio of Iron-Bearing Minerals

The total dissolved iron, divalent iron, trivalent iron, and divalent iron/total dissolved iron (Fe^2+^/Fe)) in the sulfuric acid solution (pH = 1) for coarse particles (>7.0 μm, 3.3–7.0 μm, 2.0–3.3 μm), fine particles (1.1–2.0 μm), and submicron particles (<1.1 μm) of Xuanwei coal combustion particles are listed in the Table 2. Our data showed that the total dissolved iron, divalent iron, and trivalent iron dissolved from the particles were much smaller than the dissolved amounts of raw coal and bottom ash.

The percentage of divalent iron in the total dissolved iron varied from 12 to 50% after 30 min. After 48 h of dissolution, the proportion of divalent iron in the total dissolved iron increased to 20% for the coarse particles, 30% for fine particles, and 52% for submicron particles. These data indicate that the divalent iron in the particle solution plays an important role, because Fe(II) has more oxidative potential than Fe(III).

### 3.5. Oxidation Potential of Iron

**Figure 3:**
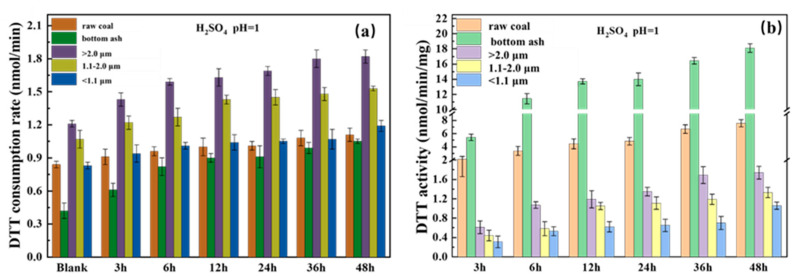
Oxidative potential of the dissolved irons of size-resolved particles from Xuanwei coal combustion after different reaction durations (H_2_SO_4_ (pH = 1), 2 g/L, 25 °C, 48 h; particle size of raw coal and bottom ash: <10 µm; the blank control group was correspondingly diluted with H_2_SO_4_ of pH = 1). (**a**) DTT consumption rate of the measured samples, (**b**) DTT activity of the size-resolved particles.

The oxidation potential of the iron-containing particles in pH 1 sulfuric acid solution at different time point is shown in the Figure 3. Compared to the blank sample, dissolved iron exhibited significant oxidative potential. The oxidation potential of the sample increased with the total dissolved iron (FeT) concentration in the particle solution (Table 2).

The DTT consumption rate (Figure 3a) of the raw coal bottom ash and the corresponding coal-fired particulate matter showed a slow increasing trend as the dissolution time extended, and the corresponding DTT activity (Figure 3b) also showed the same trend.

The oxidation of the raw coal and its bottom ash was also investigated in the study. The data showed that the dissolved iron in the bottom ash had the highest oxidation–reduction activity (DTT activity), followed by the dissolved iron in the raw coal (DTT activity after diluting the dissolved iron in the bottom ash by 100 times).

## 4. Discussion

Mineral particles in air with diameters of less than 2.5 or 1.0 micrometers could be inhaled into the lung area directly and damage human health [22].

We previously reported that there were several Fe-containing mineral particles in Xuanwei coal combustion particles, such as chlorite, illite, and kaolinite [7]. However, the toxicological effects of the iron-containing particles are still not very clear; therefore, the physicochemical characterization of the Fe-containing particles and an assessment of their oxidative potential were conducted in this study.

### 4.1. Free Radicals Generated by Fe-Containing Particles

Fe is the dominant species of transition metals in the atmosphere and is known to be associated with free radicals such as hydroxyl radicals (·OH), hydrogen peroxide (H_2_O_2_), and superoxide radicals (O_2_•−), which are created through Fenton reactions [23,24].

Fe^2+^ + O_2_ → Fe^3+^ + O_2_^−^ (7)

Fe^2+^ + O_2_^−^ → Fe^3+^ + H_2_O_2_ (8)

Fe^2+^ + H_2_O_2_ → Fe^3+^ + ·OH + OH^−^ (9)

or other ROS, such as HO_2_•:

Fe^3+^ + H_2_O_2_ → Fe^2+^ + HO_2_^•^ + H^+^ (10)

A previous report demonstrated that ·OH formation might be used as an indicator of the toxic potential of inhaled PM_2.5_. For example, greater pulmonary inflammation and oxidative stress were observed in rats who had undergone exposure to metal-rich particles [25]. Other studies reported that the toxicity associated with PM may stem from its ability to generate ·OH [26]. ·OH is believed to be the most damaging ROS. It can react rapidly with most biological molecules, ultimately damaging DNA. Therefore, ·OH generation is especially important in analyzing how human health is affected by PMs.

Our data showed that Fe^2+^ could produce a large amount of ·OH in SLF in a 24 h time period (Figure 3). The mass level of the radical increased as the size of the particles decreased, suggesting that fine and submicron particles have stronger hydroxyl radical formation ability. Additionally, the ESR data supported that there free radicals existed in Xuanwei coal combustion particles.

### 4.2. Solubility and Oxidative Potential of Iron-Containing Particles

Iron solubility is assumed to be a main factor in the evaluation of the toxicological effect of PMs. More soluble iron compounds (in an Fe^2+^ oxidation state) are assumed to be more bioavailable. The rate of iron ion release depends on iron minerology, as iron oxides react slower than iron bound in clays, increasing the conversion rate of insoluble iron to soluble iron by an order of magnitude [27].

We found that the solubility of the iron from coarse particles was lower than that of fine and submicron particles (Table 2). The main reason for this was that iron-containing particles could contain minerals such as chlorite and illite. Meanwhile, in the fine or submicron particles, iron was absorbed into the particles; therefore, the iron released easily.

The DTT consumption ratio and activity showed rapid increases followed by gradual slowing, which is consistent with the trend of iron dissolution, proving that oxidative potential falls in line with Fe concentration (Table 2). Previous data has shown that some redox chemical elements (i.e., Zn, Cd, Pb) also contributed to DTT consumption [26] (Wu et al., 2021). Therefore, these metals found in Xuanwei coal combustion particles might affect the DTT activity. This needs further study.

## 5. Conclusions

Our data showed that the iron-containing minerals accounted for more than 10% of the measured particles emitted from Xuanwei coal combustion particles. The content analysis of ·OH generated from Xuanwei coal combustion particles showed that ·OH content was dependent on the size of particles in the SLF solution after 24 h. The percentage of divalent iron released from different size particles varied: more Fe(II) could be released from the submicron particles compared with that of fine particles and coarse particles. With the mass concentration of dissolved irons increasing, the oxidative potential of the coal combustion particles increased. Armed with the above data, the toxicological mechanism of iron-containing mineral particles will be investigated further.

## Figures and Tables

**Table 1:** Chemical elements in the size-resolved particles from Xuanwei raw coal combustion (XRF) (μg/g).

	Coarse Particles			Fine Particles	Submicron Particles
	>7.0 μm	3.3–7.0 μm	2.0–3.3 μm	1.1–2.0 μm	<1.1 μm
Na	891.45	480.61	71.16	27.26	130.77
Mg	5215.94	2284.15	234.06	51.26	36.68
Al	3926.18	1818.40	278.81	49.63	369.00
P	9.48	4.95	0.45	0.41	0.86
S	2693.32	629.26	322.67	244.08	1267.11
Cl	322.44	104.05	105.17	103.73	4190.61
K	246.57	123.87	12.08	16.27	8.20
Ca	104.32	89.19	8.50	4.47	26.76
Sc	9.48	4.95	1.34	1.63	18.13
Ti	9.48	4.95	0.45	0.00	0.43
V	9.48	0.00	0.00	0.00	1.29
Cr	9.48	4.95	2.69	1.63	0.43
Mn	9.48	0.00	0.90	0.41	0.43
Fe	2976.69	2045.68	1399.80	1187.81	263.17
Co	37.93	19.82	0.45	0.00	0.43
Ni	85.35	49.55	4.48	5.70	0.00
Cu	37.93	4.95	0.45	0.41	0.43
Zn	37.93	9.91	1.79	3.66	8.63
Ga	9.48	0.00	0.00	0.41	0.43
As	9.48	0.00	0.45	0.41	5.18
Se	18.97	4.95	2.69	2.44	58.69
Br	85.35	29.73	14.77	12.61	444.09
Sr	9.48	4.95	0.45	0.00	0.86
Ba	28.45	24.77	6.71	2.03	10.36
Pb	28.45	0.00	0.00	0.00	55.67

**Table 2:** Iron dissolution (mg/L) of coarse particles, fine particles, and submicron particles in H_2_SO_4_ (pH = 1). (dissolution condition: 2 g/L, 25 °C, 48 h).

H_2_SO_4_ pH = 1		0.5 h	1 h	3 h	6 h	12 h	24 h	36 h	48 h
coarse particles (>2.0 μm)	Fe_T_	0.51	0.51	0.52	0.53	0.54	0.56	0.57	0.59
	Fe^2+^	0.06	0.06	0.07	0.07	0.08	0.1	0.11	0.12
	Fe^3+^	0.45	0.45	0.45	0.46	0.46	0.46	0.46	0.47
	Fe^2+^/Fe_T_	0.12	0.12	0.13	0.13	0.15	0.18	0.19	0.20
fine particles (1.1–2.0 μm)	Fe_T_	0.39	0.4	0.42	0.43	0.44	0.48	0.53	0.56
	Fe^2+^	0.05	0.05	0.07	0.08	0.09	0.11	0.14	0.17
	Fe^3+^	0.34	0.35	0.35	0.35	0.35	0.37	0.39	0.39
	Fe^2+^/Fe_T_	0.13	0.13	0.17	0.19	0.20	0.23	0.26	0.30
submicron particles (<1.1 μm)	Fe_T_	0.28	0.33	0.38	0.41	0.43	0.44	0.46	0.48
	Fe^2+^	0.14	0.17	0.19	0.19	0.2	0.21	0.23	0.25
	Fe^3+^	0.14	0.16	0.19	0.22	0.23	0.23	0.23	0.23
	Fe^2+^/Fe_T_	0.50	0.52	0.50	0.46	0.47	0.48	0.50	0.52

## Data Availability

Data are contained within the article.
